# Letter from Dr. Geo. N. Burwell in Reply to Prof. Flint’s Letter to Dr. Hunt, on the Subject of Pneumonia*See Art. I, in the Buffalo Medical Journal for February, 1856.

**Published:** 1856-05

**Authors:** Geo. N. Burwell

**Affiliations:** Buffalo


### BUFFALO MEDICAL JOURNAL

AND

### MONTHLY REVIEW.

**VOL. 11.** MAY, 1856. **NO. 12.**

### ORIGINAL COMMUNICATIONS.

ART. I.—*Letter from Dr. Geo. N. Burwell in reply to Prof. Flint's letter to Dr. Hunt, on the subject of Pneumonia.**

Dr. Hunt,

*Dear Sir:* The letter of Dr. Flint's, in the February number of the Buffalo Medical Journal, in reference to my strictures upon his paper on pneumonia, calls for a reply.

In the first place, it is with sincere regret that I feel obliged to notice the objectionable tone and temper of the letter, as uncalled-for by anything in my paper and out of place in a scientific discussion.

Dr. Flint writes as if he were injured by my presuming to examine his facts and opinions, and shows a sensitiveness to criticism which, I confess, astonished me to see in one so well able to take care of himself in a discussion. I was equally surprised to see him resort to a style and manner so common with those who, at fault in argument, seek by such means to hide and turn attention from their own weakness.

Dr. Flint should have remembered that this discussion was not at all of my own seeking; that criticism, "more zealous than successful," did not commence on my part; and in accepting the prominent position on one side of a controversy, and speaking of me by name and criticising my

* See Art. I, in the Buffalo Medical Journal for February, 1856.

practice (very courteously indeed, and in marked contrast with his letter) he should have anticipated a reply, received it with the same good nature that it was given, and kept to himself such offensive exhibitions of feeling and irritation as show themselves in almost every paragraph of his letter.

For two reasons I shall be careful not to imitate him; although it would be easy to retort, and the temptation to do so, I find, is going at times to be provokingly urgent. In the first place, such exhibitions of feeling always carry their own antidote; a fact popularly expressed in a homely adage, which is undoubtedly quite familiar to the Doctor, and which, therefore, I need not here quote. In the second place, I prefer now to close the discussion on my part (Dr. F. having already declined continuing it) without saying anything than I have already felt obliged to say, which would be forbidden either by courtesy or good nature.

My first stricture upon Dr. Flint's paper was, that in speaking of the pathology of pneumonia, he did not mention the important investigations of Andral and Gavarret, showing the increased quantity of fibrin in the blood in this disease. He answers that he designs yet to submit a paper on the "Diagnostic value of the buffy coat," one of the subjects referred to the committee.

I reply that the resolution calls only for the examination of the value of the buffy coat, first, in the diagnosis of pneumonia from other diseases; and, second, as a means of distinguishing the different varieties of pneumonia from each other. This, it will readily be seen, does not necessarily comprehend the whole subject of the variations of fibrin in the blood in the different forms in which pneumonia presents itself. My stricture, therefore, holds good.

I wish, in this connection, to refer to the investigations of Rokitansky upon this important point in the pathology of pneumonia. It is certainly gratifying to me to find that the conclusions to which I arrived, from the examination of the blood drawn during the course of the disease (opinions which I have held, in part, for eight or ten years past) have received a general confirmation by Rokitansky from the examination, after death, of the clots in the heart and great vessels, and the depositions or exudations into the lungs.

In the first place, he every where teaches the great doctrine of the humoral pathology of pneumonia. In the second place, he shows that it is the *quality* of the fibrin that determines the kind or type of the inflammatory action.

He describes two leading forms of fibrin: the *first*, plastic and organizable, and possessing the property of adhesiveness; the *second*, not organizable, with a tendency to disintegration, and possessing "little adhesive property."* There are two varieties of each of these forms.

The reader of the cases and papers on this subject which I read to the society the past summer and fall, will find in them these general ideas (with one exception) of the humoral pathology of pneumonia and of the variations in the tenacity and adhesiveness of the blood clot fully expressed.

This exception is, that I recognised but one kind of fibrin, viz., an organic substance characterized by tenacity, elasticity and firmness of structure (corresponding to the simple plastic organizable fibrin of Rokitansky) while this pathologist makes a second variety *distinguished by depravation of quality;* and which he calls croupous fibrin. Paget, I see, uses the term fibrin as I do, and the depraved or croupous fibrin of Rokitansky, he calls corpuscular lymph.

Now this plastic fibrin I find to characterize sthenic pneumonia. Its deposition in the lungs in the pneumonia of Vienna, is only "occasionally" seen;† so rarely, indeed, that Rokitansky, in describing the pneumoniæ, does not make a separate variety corresponding with this form of fibrin, although he frequently calls attention to the importance of understanding the differences presented by the inflammatory product in regard to its plasticity.

Those cases of pneumonia in which the blood had not this tenacity or adhesiveness, I proposed to call congestive pneumonia. Rokitansky calls them croupous pneumonia, a name which corresponds with the croupous or depraved variety of fibrin which characterizes them. To this class he says "almost all the pneumonia [of Vienna] belong."‡

I could not speak, from my own observations, of the character of the blood in typhoid pneumonia, never having bled a case; but I expressed "a strong assurance" that it would not have the normal quantity of fibrin. What is Rokitansky's evidence upon this point? He says, "genuine typhus [typhoid] pneumonia, does not develop a fibrin crasis;"‖ and again, that the blood in this form of disease, "is characterized by a dirty brownish red or chocolate colored, very yielding inflammatory product, which soon breaks down when there is great disease of the blood, and extreme absence of plasticity."§

* Rokitansky's Pathological Anatomy, vol i., pp. 83, 84.

† Ibid, vol. i., p. 110.

‡ Ibid, vol. i., p. 112.

‖ Ibid, vol. i., p. 276.

§ Ibid, vol. iv., p. 97.

Am I not pretty well sustained in my observations and generalizations upon this most important point in the pathology of pneumonia?

My second stricture was upon the statement of fact made by Dr. Flint, that "the inflammation, in the great majority of cases, extends over an entire lobe at least." I opposed to this the fact that in one only of the eight cases I saw last summer, did the inflammation involve an entire lobe; and I made the general statement that "it is very rarely indeed that I see an entire lobe affected." In making it, I, of course, had reference only to cases which came early under my own treatment.

On reviewing this statement of my own experience, I must insist upon its accuracy. As far as it depends upon the eight cases already referred to, there can be no mistake of observation. All were seen and examined by other gentlemen than myself. As far as it depends upon my remembrance of other than the eight cases, there are, undoubtedly, chances for error; but to the best of my recollection I have rarely seen an entire lobe affected in cases I have treated from the commencement of the attack: a result which I attribute mostly to my habit of early and, when necessary, repeated venesection.

Dr. F. makes the following extract from Grisolle, with the double object of sustaining his own statement and of throwing doubts upon the accuracy of my observations, viz., in 132 patients, "the inflammation which had begun by affecting only a sixth, or a fifth, or a quarter of the pulmonary surface, extended more or less rapidly over a half, or two-thirds, or the whole of the organ."

In the first place, this statement is far from affirming that the inflammation, in the vast majority of instances, not only does involve the entire lobe in which it commenced, but also that it is limited to that lobe; neither does it affirm that it is impossible, by early and judicious bloodletting, to limit the diffusion of the inflammation. Grisolle could not mean to be thus understood, or he would not be likely to deem "bloodletting so important a therapeutical measure in this disease that he could only conscientiously withhold it in a very few peculiarly mild cases."

In the second place, the fact of the limitation of the inflammation to a portion only of a lobe in the cases I reported as such, is too well confirmed by the observation of other gentlemen than myself, to be doubted. Explain it or contrast it with other statements as you choose, the fact remains just as I have stated.

Dr. F. wishes to know how I determine whether an inflammation is traveling inwardly. It is by examining other portions of the lung than the place first affected. In a pneumonia commencing on the posterior surface of the lower lobe of one of the lungs, I examine the axillary and frontal regions of that lobe, and if I do not find any evidences of pneumonia in either of these regions at any time during the course of the disease, I conclude that the inflammation has not extended far inwardly. If in pneumonia of the anterior surface of an upper lobe I find no evidences of it posteriorly, I draw the same conclusion.

Seldom seeing, in my own practice, an entire lobe affected, it is not common for me to see the inflammation extend to a second lobe. I nowhere said that the interlobular fissure had no effect to retard the extension of a pneumonia to an adjoining lobe. On the contrary, my observations entirely accord with Grisolle's, that where different lobes are affected it is at different periods of the disease. A beautiful confirmation of this law occurred to me last fall in a pneumonia (of the congestive form and not bled) which commenced at the upper part of the lower lobe of the left lung, spread to the base of the lung "like the march of an erysipelas," and then on the sixth day of the disease overleaped the fissure and spread over the posterior surface of the upper lobe. It was interesting to observe in this case, that the axillary and sub-clavicular regions remained intact; the inflammation must, therefore, have been confined to the posterior surface of the two lobes—it could not have extended far inwardly into the substance of the lung. It will be noticed, also, that the interlobular fissure delayed, for four or five days, the extension of the inflammation into the upper lobe.

Again, the effect of the interlobular fissure in preventing the diffusion of the inflammation, is shown in those cases, sufficiently frequent to be familiar to all, in which the disease commences in the upper posterior part of a lower lobe, and extends gradually downward to the base of the chest; but not upward into the upper lobe.

Having shown how easy it is to determine, during life, whether or not an entire lobe be inflamed, I cannot accept of Grisolle's autopsies to determine the point in dispute. Besides it is easy to understand why, in post-mortem examinations, the implication of the entire lobe should be found to be the rule rather than the exception.

I justify my apprehensions for my patient's safety when an entire lobe is affected, by my experience the past year. During that period I have seen an entire lobe inflamed four times. The first was the case of the boy, McMichael, whose case is reported in the Buffalo Medical Journal for October 1855, page 276. He was saved, I believe, only by free and extraordinary bloodletting. The second case, Catharine McGinn, is reported in the same number of the Journal, page 286. No one who saw her could doubt her great danger. The third was the case of a boy with sthenic pneumonia. He had the advantage of one good bleeding, and, in my judgment, should have been bled again. He recovered only at the end of two months. The fourth is the case of a girl, 19 years of age, I lately saw in consultation, at the end of the fifth week of her sickness. Percussion was flat over the whole posterior and axillary regions of the lower lobe of the left lung, with a correspondingly well-developed bronchial respiration. Some weeks must elapse before her restoration to health, if she is fortunate enough to escape purulent liquefaction of the exudation. She had not been bled. Exclude the first case (in which the restoration was rapid and complete) and I think the list rather a sorry one.

My third stricture was upon the statement of fact "that double pneumomia was exceedingly rare in the adult."

In quoting Grisolle's statistics of cases of double pneumonia, I had not the advantage of referring to his own work. I applied for it unsuccessfully, at the bookstores, here, and (through our booksellers) in New York. The statement I made may be found in Dr. Wood's work on the Practice of Medicine, vol. ii., page 6, edition of 1852. It will be seen that Dr. Wood does not give any intimation that the statistics are not in accordance with Grisolle's own opinions. An examination of authorities upon the question of the frequency of double pneumonia, will show a balance largely against Grisolle. He and Barthe place it at about *one-sixteenth* of all cases: while the authorities he quoted, place it at *nearly one-fifth.* Dr. Stokes (certainly one of the most reliable of auscultators) places it at rather *over one-fifth;** and Andral at *one-ninth.*†

Dr. Flint thinks I have seen a great many cases of chronic pneumonia (four cases in eighteen months, while Grisolle—up to the time his work was written—had met with one case only.) Dr. F. does not seem to know that the term chronic, as applied to pneumonia, is used in two significations: one in contradistinction to the term acute, and the other as applied to a peculiar and very rare form of this disease. Had he been aware of this, the reading of the cases which I classified under the head of chronic pneumonia, would have shewn him in which sense I used it. Rokitansky uses the term

---

"chronic pneumonia" as I do. He says that pneumonia "sometimes runs a chronic course, being either uniformly prolonged in all its stages, *or one or other of them being especially protracted.*" "It is totally different from the affection which we commonly find described in pathological treatises as chronic inflammation of the lungs."* That form of pneumonia to which Dr. F. would restrict the term chronic, Rokitansky calls interstitial pneumonia.

My fourth stricture was upon the propriety of calling (which I supposed Dr. Flint to do) the consecutive inflammation of the second lung a complication.

Dr. F. made the broad statement that of thirty-eight cases he had not had an instance of death from "primitive *uncomplicated* pneumonia." To arrive at this conclusion he excluded *two* cases of death in which no autopsy was made; *one* case of death from complicated pneumonia, and *three* cases of death from double pneumonia.

These last, he said, were excluded because "double pneumonia is a dangerous form of disease." Now it is *so* easy to prove any disease mild and self-limited, if we be allowed to exclude all the dangerous cases, that in giving this as a reason, I really thought it must have been a slip of the pen, in the hurry of composition; and in that case I was, of course, obliged to adopt the opinion that they were excluded as cases of complicated pneumonia. But he does not allow this interpretation of his opinion; and I cannot allow exclusions for such a reason. His statement, then, needs amending.

Dr. Flint misapprehends me in thinking that I impute to him the discovery that "primary idiopathic pneumonia, disconnected from complications, associated morbid conditions, and incidental circumstances intrinsically tends to recovery." Ready at all times to accord him every credit for what he has or may yet do for medicine, I did not attribute to him the originality of this idea. I had made the statement that pneumonia, in its totality, was one of the most fatal of diseases. Instead of combating this great fact, Dr. F. substituted the above statement, which I called a "new position," (meaning) in our discussion.

*Of the Vienna Statistics.* In my remarks upon these statistics I acknowledged their value; and I expressed the opinion that were the cases correctly classified "a majority" of them would be found to belong to what I call the congestive variety of pneumonia: that is to a class belonging neither to the

---

* Rokitansky's Pathological Anatomy, vol. iv., p. 72.

typhoid nor to the active sthenic varieties of the disease; an opinion perfectly logical, I insist, when we consider their light mortality in connection with the entire absence of any controlling treatment. Now where does Rokitansky place the pneumonia of Vienna? In the croupous class; the class characterized by fibrin of small adhesiveness and which corresponds, in part, with my idea of congestive pneumonia. To this class he affirms "almost all the pneumonia [of Vienna] belong."*

My views of the pathology of pneumonia, were not formed "a priori," leaving for future accumulation facts in support of them. Had I kept a careful record of my experience with this disease, for the last dozen years, I should now have written proof in support of my opinions, and the ability fully to describe congestive pneumonia from recorded cases. But like most physicians fully engaged in the practice of medicine, I have neglected keeping any general record of my cases; and now, when my views are called in question, and a defence of them forced upon me, I cannot cite my past unrecorded experience as proof to others, although it justifies to myself my own opinions. I had, therefore, to ask time to collect evidence of the correctness of my views. Fortunately the observations and experiments of Rokitansky and Paget, of the opinions of neither of whom did I have a knowledge at the time of writing the paper I read at the November meeting of the society, will save me from that labor. I would refer the reader to the works of these observers for proofs of the connection between the constitution of the blood and most internal inflammations.

Before leaving the subject of congestive pneumonia, I would here respectfully ask the consideration of the Profession to the propriety of styling by this name, those cases of this disease in which the elements of congestion predominate over those of inflammation. Many such cases are constantly occurring in large cities, in hospitals and in malarial districts, which recover by rest in bed, and without any controlling treatment, other than is called for by the morbid condition complicating them. It seems to me to be a misnomer to call such cases by the more formidable name of acute sthenic pneumonia.

*Of the British Army and Navy Statistics, and Dr. Ames' cases.* Dr. Flint's argument was as follows: The mortality from pneumonia in the British Army and Navy is exceedingly light; *therefore* uncomplicated [sthenic] pneumonia will almost uniformly get well without bloodletting. I

* Rokitansky's Pathological Anatomy, vol. i., p. 110.

hinted to him that the English practice in this disease, is early and free venesection, and that to perfect his argument he must show that their own practice was not adopted in their own army and navy. If bleeding was generally practiced, of which there is every probability, the citation would certainly not have any value as proof of the uniform tendency of sthenic pneumonia to recover without bloodletting. Did I ask what was unreasonable? Have I not a right to insist upon my demand before admitting the citation as evidence?

Again, Dr. F. argued thus: Dr. Ames' cases of epidemic pneumonia prevailing in a malarial district, get well under the use of quinine; *therefore* sporadic [sthenic] pneumonia will almost invariably recover without bleeding. But no such conclusion can logically follow the proposition. The mere statement of the argument shows its fallacy.

The influence of malaria upon the course and symptoms of pneumonia, does not appear to have occurred to Dr. F. I would refer him to an article in the October, 1855, number of the Buffalo Medical Journal, page 266, on pneumonia and malaria, by Dr. Morse, of Bradford, Steuben Co.; and also to the excellent article on the same subject, in the November number, page 340, by Dr. Wm. Van Pelt. Would Dr. F. treat the pneumonia prevailing on the hills of Springville, in the same way he would that prevailing in the Tonawanda swamp?

Dr. Flint is not satisfied with my objections to his comparison of the six cases treated by him, with the four I had the honor of reporting to the society in July last; nor with my reasons for considering his cases to be of a milder type than mine.

I could state other and stronger reasons why our respective sets of cases belong to different varieties of the disease; but it is not necessary I should do so until he has first made an attempt to show them to be of one and the same type.

Very respectfully,

BUFFALO, March, 1856. GEO. N. BURWELL.

